# Prevalence and social drivers of HIV among married and cohabitating heterosexual adults in south-eastern Tanzania: analysis of adult health community cohort data

**DOI:** 10.3402/gha.v8.28941

**Published:** 2015-09-30

**Authors:** Sally M. Mtenga, Constanze Pfeiffer, Sonja Merten, Masuma Mamdani, Amon Exavery, Joke Haafkens, Marcel Tanner, Eveline Geubbels

**Affiliations:** 1 Ifakara Health Institute, Ifakara, Tanzania; 2 Swiss Tropical and Public Health Institute (Swiss TPH), Basel, Switzerland; 3 Department of Epidemiology & Public Health (EPH), University of Basel, Basel, Switzerland; 4 Centre for Social Science and Global Health, Amsterdam Institute of Advanced Labour Studies, University of Amsterdam, Amsterdam, The Netherlands; 5 Department of General Practice AMC, University of Amsterdam, Amsterdam, The Netherlands

**Keywords:** HIV, couples, social drivers, community surveillance data, Tanzania

## Abstract

**Background:**

In sub-Saharan Africa, the prevalence of HIV among married and cohabiting couples is substantial. Information about the underlying social drivers of HIV transmission in couples is critical for the development of structural approaches to HIV prevention, but not readily available. We explored the association between social drivers, practices, and HIV status among stable couples in Ifakara, Tanzania.

**Design:**

Using a cross-sectional design, we analyzed data from a sample of 3,988 married or cohabiting individuals, aged 15 years and older from the MZIMA adult health community cohort study of 2013. Sociodemographic factors (sex, income, age, and education), gender norms (perceived acceptability for a wife to ask her partner to use a condom when she knows he is HIV positive), marriage characteristics (being in a monogamous or a polygamous marriage, being remarried), sexual behavior practices (lifetime number of sexual partners and concurrent sexual partners), health system factors (ever used voluntary HIV counseling and testing), and lifestyle patterns (alcohol use) were used to explore the odds of being HIV positive, with 95% confidence intervals.

**Results:**

Prevalence of HIV/AIDS was 6.7% (5.9% males and 7.1% females). Gender norms, that is, perception that a woman is not justified to ask her husband to use a condom even when she knows he has a disease (adjusted odds ratio AOR=1.51, 95% CI 1.06–2.17), marital characteristics, that is, being remarried (AOR=1.49, 95% CI 1.08–2.04), and sexual behavior characteristics, that is, lifetime number of sexual partners (2–4: AOR=1.47, 95% CI 1.02–2.11; 5+: AOR=1.61, 95% CI 1.05–2.47) were the main independent predictors of HIV prevalence.

**Conclusions:**

Among married or cohabiting individuals, the key social drivers/practices that appeared to make people more vulnerable for HIV are gender norms, marriage characteristics (being remarried), and sexual behavior practices (lifetime number of sexual partners). Married and cohabiting couples are an important target group for HIV prevention efforts in Tanzania. In addition to individual interventions, structural interventions are needed to address the gender norms, remarriage, and sexual practices that shape differential vulnerability to HIV in stable couples.

Sub-Saharan Africa (SSA) remains the region with the highest prevalence of HIV infections worldwide (1). More than half of the adults in SSA are living in stable marital or cohabiting heterosexual relationships (2). The HIV prevalence among married and cohabiting couples is substantial (2–4). Moreover, close to two-thirds of the new HIV infections occur in stable married or cohabiting couples (2). Consequently, married and cohabiting heterosexuals constitute an important target population for HIV prevention efforts in the region.

Transmission of HIV within couples can be reduced by individual interventions such as voluntary HIV counseling and testing (VCT), condom provision, and early antiretroviral treatment (5–9). There is increasing recognition, however, that these HIV prevention efforts cannot succeed in the long term without structural approaches that address the societal or social drivers which shape people's vulnerability for HIV (10–13). According to Auerbach et al., the concept ‘social drivers’ refers to ‘the core social processes and arrangements – reflective of social and cultural norms, values, networks, structures, and institutions – that operate around and in concert with individual behaviors and practices to influence HIV epidemics in particular settings’ (11). As such, according to these authors, the concept social drivers shows similarity with what has been referred to as ‘social determinants’ in the World Health Organization framework of social determinants of health (14). In this paper, we will use the concept social drivers as used by Auerbach et al. Research on social drivers of HIV in Africa is still in its infancy and results are often inconclusive (15). However, a number of social drivers were found to be linked to the inequitable distribution of HIV prevalence within or between various populations in this region.

Besides biological sex (HIV rates are higher in women than in men) (16, 17), some of the previously identified social drivers that may increase the vulnerability for HIV infections include social and demographic factors (e.g. poverty and level of education) (17), gender inequality (18–22), sexual behavior practices (e.g. the number of lifetime and concurrent sexual partners (23) or the lack of male circumcision (24–26), and health system factors (e.g. limited access to HIV prevention or treatment programs).

In Tanzania, evidence on social drivers influencing the differential distribution of HIV among individuals living in stable relationships is still limited, and mainly based on qualitative studies among married women (27–30). To facilitate the development of more comprehensive and structural approaches to HIV prevention for heterosexual couples, the existing evidence base needs to be completed with quantitative data.

In this paper, we analyzed data from a community health cohort study conducted in the Ifakara region in Tanzania in 2012–2013. To help inform structural approaches to HIV prevention, our analysis focused on married or cohabiting men and women in the cohort and we assessed how a number of previously identified social drivers of HIV were associated with the prevalence of HIV in this group.

We hope that this data will provide additional information to inform HIV programmers on factors that need to be addressed to prevent HIV in stable couples.

## Methods

### Context of the study

#### Marriage in Tanzania

During the early postcolonial period, customary and Islamic law governed the area of family law in Tanzania. In 1971, Tanzania adopted the Law of Marriage Act (LMA) (31). This act integrated customary and Islamic law into civil law but provided women with (somewhat) better civil rights upon marriage and divorce.

The LMA defines marriage as ‘the voluntary union of a man and a woman, intended to last for their joint lives’. Minimum age requirements for marriage are 18 for men and 15 for women. A marriage may either be monogamous or polygamous. Polygamous marriages are only allowed to men. A man has the legal right to change the marriage contract from monogamous to polygamous or vice versa, but only with the wife's consent. Polygamous relationships in Tanzania can also include ‘unofficial’ relationships, whereby men marry one woman by statutory law but form extra-legal domestic and sexual unions with other women. Called ‘unofficial’ or ‘secondary’ co-wives, these women are *de facto* married in that they have regular sexual intercourse with only one man, are financially maintained by him and have children whose paternity he acknowledges (31, 32). About a quarter of the women in Tanzania live in polygamous marriages (32). The LMA also provides separation and divorce provisions and rules for maintenance of women and children upon separation or divorce. Although the law was seen as a milestone in the fight for women's rights in Tanzania in 1971, it has also been widely criticized for maintaining certain gender discriminatory social practices such as polygamy for men and different marriage ages for men and women (32).

#### The Ifakara MZIMA adult health community cohort

This study uses data from individuals in married or cohabiting partnerships who participated in the MZIMA community health cohort study (33). The MZIMA study is a repeated population-based household survey, involving a representative cohort of persons aged 15 years and older from the Ifakara region.

The MZIMA study was set up to identify the prevalence, incidence, and determinants of non-communicable diseases and HIV over time, as well as health-seeking behavior of the affected population in Ifakara town, administrative town of the Kilombero district of the Morogoro region in Southern Tanzania.

Data were collected in two areas of the Ifakara Urban Health and Demographic Surveillance System located in Ifakara town, namely the villages Viwanja Sitini and Mlabani (34).

### Study design and data collection

This study has cross-sectional design, using data from questionnaires and HIV tests from the MZIMA study. The fieldwork for first round of the MZIMA study, which took place between April 2012 and April 2013, was conducted by a team of 18 research assistants, 3 field supervisors, 5 professional counselors, 7 professional nurses, 3 clinical officers, 1 laboratory technician, 1 medical sociologist, 1 epidemiologist, 1 statistician, and 1 community liaison officer. Prior to the field work, the field team received a 2-week training. The training was meant to inform the team members about the design and the purpose of the MZIMA community health cohort study; their specific roles; and on how to implement the study tools and standard operating procedures, data quality assurance measures, and appropriate research ethics. Field team members identified participants and collected baseline data during household visits. During the first household visit, the research team asked household heads for their initial consent to carry out research activities in his/her premises. Later on, individual informed consent was also obtained from household members who were eligible for the study. Separate consent was obtained for the provision and storage of verbal survey data, blood specimens, and for conducting HIV tests on those specimens.

Using standardized questionnaires, field workers held face-to-face interviews in the local Kiswahili language to collect data on health, sociodemographic, and behavioral characteristics including sexual behaviours of the selected study participants. Open Data Kit software in tablet computers was used to support the collection and entering of the data. Subsequently, professional nurses, who were trained for this task, took venous blood samples from participants using a Vacutainer needle.

Finally, all participants were offered the possibility of receiving voluntary counseling and testing, according to the national guidelines (35). Interviews were held before the blood tests and HIV counseling sessions to avoid social desirability bias.

The collected blood specimens were stored in EDTA Vacutainer tubes. Immediately after each research visit, these tubes were transported in cooler boxes to the main Ifakara Health Institute laboratory, which is located about 1 km from the field sites.

Questionnaire data were checked through a series of internal consistency and range checks to identify any illogical responses. Questionnaire results and blood samples were linked to an individual through unique identification numbers.

**Fig. 1:**
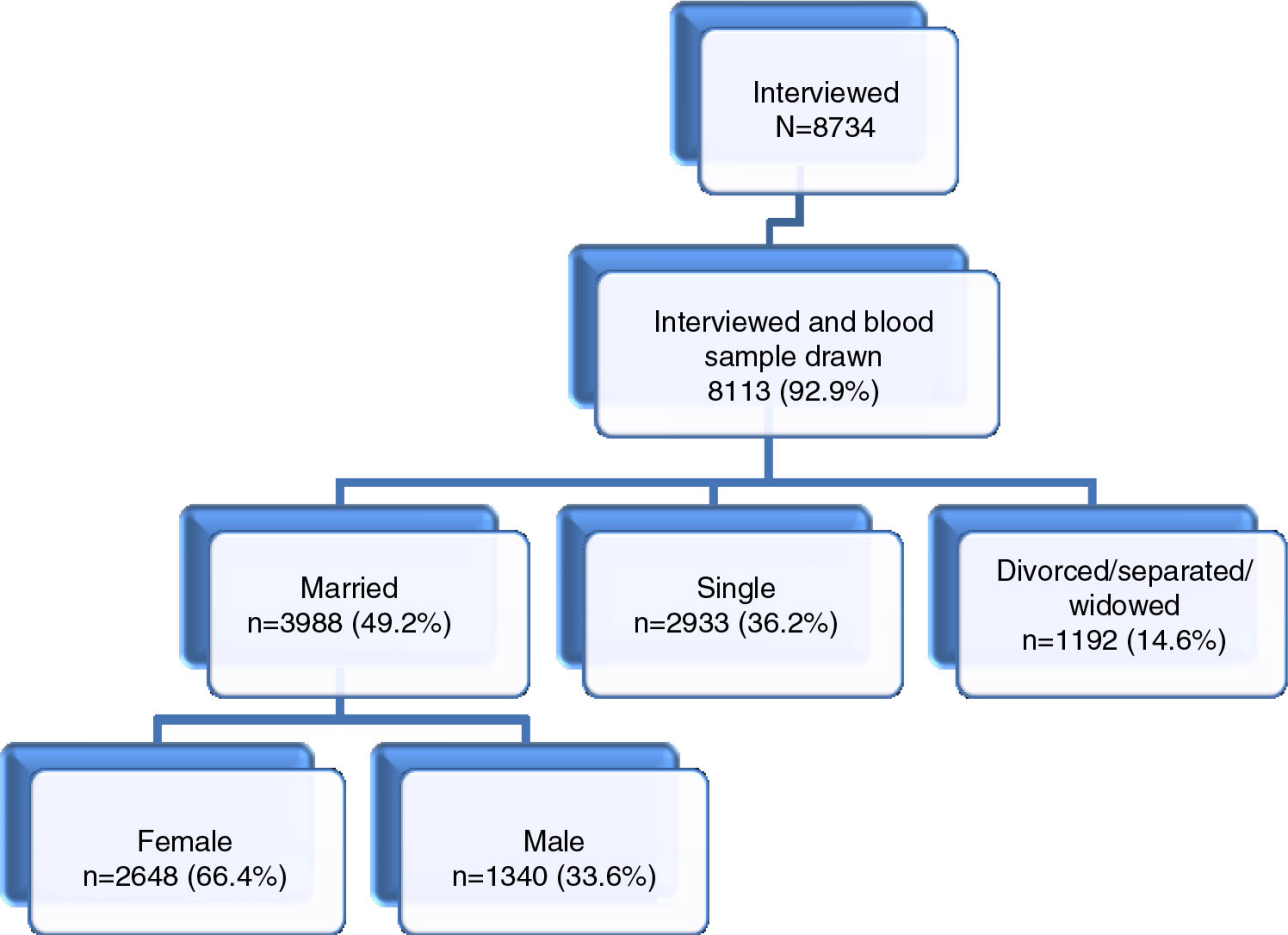
Participant flow diagram.

During the first round of the MZIMA cohort study 8,734 were interviewed, 92.9% of whom provided blood samples for HIV testing (Fig. 1). Participants in the cohort were defined as being married or cohabiting if they had indicated on the questionnaire that their marital status was ‘officially’ married or cohabiting. This corresponds to the definition of marriage/cohabitation in the LMA (31). Because we aimed to investigate factors that make people in stable relationships vulnerable for HIV, in the present study we only included data from the MZIMA cohort participants who said they were officially married or cohabiting (49.2%).

### Variables and measures

Box 1 describes the main variables used in the present study. HIV status was the main outcome or dependent variable. To establish HIV status laboratory technicians tested all blood samples for HIV-1 antibodies using two consecutive Elisa tests (Vironostika HIV Ag/Ab antigen/antibody and Vironostika HIV Uni-Form II plus 0, ELISA; Vironostika^®^, Biomérieux BV, Boxtel, The Netherlands). Samples with two positive tests were considered HIV positive. HIV status was defined as a binary variable, with 1 indicating HIV positive and 0 indicating HIV negative.

***Box 1.***Variables evaluated in the analysis

**Table:** 

Dependent variable	HIV positive (yes/no)
Independent variables	Social and demographic characteristics: sex (men/women) age (four age categories) religion (Muslim, Christian-Catholic, Christian-other, and other) ethnicity (North- Western, North- Eastern, and Southern) employment (being engaged in income generating activity, yes/no) education (received formal education, yes/no) Gender norms: ‘a woman can ask her husband to use a condom when she know he has a disease’ (yes, no) Marriage characteristics: ‘being in a polygamous in or a monogamous marriage’ (yes/no) ‘being in a first or subsequent (second) marriage’ (yes/no). Sexual behavior characteristics: number of lifetime sexual partners (1, 2–4, ≥5) ‘used condom at first sex’ (yes/no). concurrent partnership (yes/no) Health care utilization: ‘ever received VCT’ (yes/no). Lifestyle characteristics: ‘ever drank alcohol’ (yes/no)

Based on a review of previous literature on social drivers of HIV (12, 13, 15–17, 23) and the WHO social determinants of health framework (14) we selected six groups of items from the MZIMA self-report questionnaire as independent predictor variables for analyzing the impact of social drivers on HIV status in couples: 1) social and demographic characteristics; 2) gender norms; 3) marriage characteristics; 4) sexual behavior characteristics; 5) health care utilization characteristics; and 6) lifestyle characteristics. The selected variables for group 1 refer to sociodemographic characteristics which are fixed and not directly amenable to change through interventions. We included these variables in this study to provide background information on the study population. The selected variables for groups 2–6 refer to social practices/drivers that are ‘socially constructed’ and may therefore be potentially relevant for the development of structural interventions to prevent HIV.

### Statistical analyses

All married or cohabiting individuals from the MZIMA cohort with complete data for interviews and HIV status were included in the analysis (Fig. 1). The analysis sample for this study consisted of 3,988 individuals of age 15 years and older, 267 of whom had (positive) HIV outcomes. This is sufficient to perform multivariable statistical analysis with adequate statistical power, when using Peduzzi's (36) rule of needing at least 10 outcome events per covariate included in the multivariable model.

All analyses were performed using STATA version 11 (StataCorp, USA). Descriptive statistics were used to list outcomes for all variables. Then bivariate analyses (cross tabulation) were conducted to analyze the associations between the dependent variable – HIV status – and each of the independent variables.

The strengths of the associations between the dependent and independent variables were tested using the Pearson chi-square tests, because all variables were categorical. Associations were regarded statistically significant if the *p*-value was <0.05. Finally, multivariate logistic regression analysis was performed to calculate the adjusted odds ratios (AOR) and 95% confidence interval (CI), to identify independent predictors of HIV infection among the study population.

The model used sex and level of education as fixed *a priori* covariates in all models. Selection of the other variables for the multivariate analyses was based on their ability to improve the overall model using log likelihood ratio test (37). Statistical interactions between variables of interest were also assessed. Odds ratios, their corresponding 95% confidence intervals, and *p*-values were reported for the final model.

## Ethical statement

The MZIMA surveillance study was approved by the Tanzanian Medical Research Coordinating Committee (approval number NIMR/HQ/R.8a1Vol. IX/I320) and by the Ifakara Health Institute Review Board (approval number IHI/IRB/AM/ 01- 2014). Participants were asked to indicate their informed consent by signing or providing their thumb finger print in the presence of a witness, after they had read and understood the contents stipulated in the informed consent form.

## Results

### Participant's characteristics

Table 1 describes the characteristics of study participants. One-third (33.6%) were male and two-thirds (66.4%) were female. Although all participants lived in Ifakara town at the time of study, most (89%) participants reported to have originated from the southern parts of the country.

**Table 1:** Characteristics of the study population^a^: frequency distribution (*n=*3,737)

Variable	Number of respondents (*n*)	%
Total	3,988	100.0
Social and demographic characteristics		
Sex		
Male	1,340	33.6
Female	2,648	66.4
Age (years)^b^		
≤20	175	4.4
21–30	1,265	31.8
31–40	1,159	29.1
41–50	613	15.4
50+	770	19.3
Mean=38.4, SD=14.5, Min=13, Max=99	–	–
Performs any income-generating activity		
Yes	2,909	72.9
No	1,079	27.1
Ever had formal education		
Yes	3,494	87.6
No	494	12.4
Religion		
Muslim	1,559	39.1
Christian-Catholic	2,087	52.3
Other Christian	322	8.1
Other/none	20	0.5
Ethnic group		
Northern Western	307	7.7
Northern Eastern	133	3.3
Southern	3,548	89.0
Marital characteristics		
Marital status		
Polygamous	104	2.61
Monogamous	3,884	97.39
Remarried		
Yes	674	16.9
No	3,314	83.1
Sexual behavior characteristics		
Lifetime number of sexual partners^b^		
1	901	24.1
2–4	1,837	49.2
5+	999	26.7
Mean=4.6, SD=7.2, Min=1, Max=100	–	–
Condom use at first sex^b^		
Yes	525	13.3
No	3,384	85.8
Don't know	33	0.8
Concurrent partnerships		
No	3,719	93.3
Yes	269	6.7
Gender norms		
Beliefs a woman is justified to ask their husbands to use a condom if she knows he has a disease)		
Yes	3,454	86.6
No	534	13.4
Health care utilization		
Ever received VCT		
Yes	1,008	25.3
No	2,980	74.7
Lifestyle characteristics		
Ever drank alcohol		
Yes	1,103	27.7
No	2,885	72.3

a Currently married and cohabitating heterosexuals adults who participated in MZIMA adult health community cohort conducted in Ifakara, Tanzania.

b Missing data for some respondents.

The mean age of the participants was 38.4 (±14.5) years, ranging from 14 to 99. The majority (87.6%) had received a formal education and 72.9% were performing income-generating activity. More than half (52.3%) of participants were Christians, whereas 39.1% were Muslims. Most participants (97.39%) were living in a monogamous relationship.

### HIV positive status by background characteristics

As shown in Table 2, the overall HIV prevalence among married and cohabiting individuals in the MZIMA cohort was 6.7%. Although rates were lower among men than among women (5.9% vs. 7.1%), this sex difference was not statistically significant (*p*<0.163). HIV infection rates were higher among individuals who were remarried than among those who were living with their first partner (9.8% vs. 6%, *p*<0.001). Statistically significant differences were not found for any of the other variables. However, HIV rates were higher among individuals who had more than one sexual partner during their life, as compared to those who had not (7.2% vs. 4.9%, *p=*0.053). Similarly, higher rates were found among study participants who believed that a woman is not justified to ask her husband to use a condom if she knows he has a disease, as compared to those who did not (8.4% vs. 6.4%, *p=*0.080).

**Table 2:** Prevalence of HIV by background characteristics and cross tabulation (*n=*3,737^a^)

Variable	% HIV positive	*p*
Overall	6.7	–
Social demographic characteristics		
Sex		
Male	5.9	0.163
Female	7.1	
Age (years)		
≤20	4.6	0.473
21–30	6.7	
31–40	7.4	
41–50	7.0	
50+	5.7	
Performs any income-generating activity		
Yes	6.8	0.571
No	6.3	
Ever had formal education		
Yes	6.4	0.081
No	8.5	
Religion		
Muslim	7.3	0.618
Christian-Catholic	6.2	
Other Christian	6.5	
Other/none	5.0	
Ethnic group		
Northern Western	7.5	0.514
Northern Eastern	4.5	
Southern	6.7	
Marital characteristics		
Marital status		
Polygamous	2.8	0.117
Monogamous	6.7	
Remarried		
Yes	9.8	<0.001
No	6.0	
Sexual behavior characteristics		
Lifetime number of sexual partners		
1	4.9	0.053
2–4	7.2	
5+	7.2	
Condom use at first sex		
Yes	6.1	0.245
No	6.9	
Don't know	0.0	
Concurrent partnerships		
No	6.9	0.079
Yes	4.1	
Gender norms		
Beliefs that a woman is justified to ask their husbands to use a condom if she knows he has a disease		
Yes	6.4	0.080
No	8.4	
Health care utilization		
Ever had VCT		
Yes	7.8	0.086
No	6.3	
Lifestyle characteristics		
Ever drank alcohol		
Yes	7.3	0.362
No	6.5	

a Currently married and cohabitating heterosexuals adults who participated in MZIMA adult health community cohort conducted in Ifakara, Tanzania.

The prevalence of HIV was as low as 4.1% among individuals with concurrent partners and as high as 6.9% among individuals with no concurrent partners, (*p=*0.079). Moreover, higher HIV rates were observed among people with a lower educational level (6.8%) than higher levels of education (6.3%) (*p=*0.081). Disaggregated data for men and women are provided in Supplementary Tables 1a and 2a.

### Factors associated with HIV infection among married and cohabiting individuals

Table 3 presents results from the multivariate model of correlates of HIV positive status. The odds of being HIV positive were 49% higher among participants who were remarried as compared to those who never remarried (OR=1.49, 95% CI 1.08–2.04).

**Table 3:** HIV positive status by background characteristics: multivariate logistic regression (*n=*3,737)^a^

Variable	Odds ratio (OR)	95% Confidence interval (CI)	*p*
Social demographic characteristics			
Sex			
Male (ref)	1.00	–	–
Female	1.23	0.90–1.67	0.189
Ever had formal education			
Yes (ref)	1.00	–	–
No	1.31	0.90–1.90	0.153
Marital characteristics			
Remarried			
No (ref)	1.00	–	–
Yes	1.49	1.08–2.04	0.014
Sexual behavior characteristics			
Lifetime number of sexual partners			
1 (ref)	1.00	–	–
2–4	1.47	1.02–2.11	0.038
5+	1.61	1.05–2.47	0.028
Gender norms			
Woman's status (believing that a woman is justified to ask their husbands to use a condom if she knows he has a disease)			
Yes (ref)	1.00	–	–
No	1.51	1.06–2.17	0.024
Health care utilization			
Ever had VCT			
Yes (ref)	1.00	–	–
No	0.76	0.57–1.01	0.061

Ref=reference/baseline category.

a Currently married and cohabitating heterosexuals adults who participated in MZIMA adult health community cohort conducted in Ifakara, Tanzania.

Similarly, gender norms were also associated with HIV prevalence; HIV rates were higher among respondents who believed that a woman is not justified to ask her husband to use a condom if she knows he has a disease as compared to those who did not believe this (OR=1.51, 95% CI 1.06–2.17). Sexual behavior characteristics were also associated with HIV prevalence; the odds of being HIV positive increased with the reported number of lifetime sexual partners. Participants who reported 2–4 lifetime sexual partners were more likely to be HIV positive as compared to those who had only one lifetime sexual partner (OR=1.47, 95% CI 1.02–2.11). The odds rose even further in those reporting five or more lifetime sexual partners (OR=1.61, 95% CI 1.05–2.47). Although females were 23% more likely to be HIV positive than males, biological sex was not a statistically significant predictor of HIV (OR=1.23, 95% CI 0.90–1.67).

## Discussion

This study contributes to our understanding of social drivers of HIV among legally married or cohabiting heterosexual adults in Tanzania. The HIV prevalence rates for married or cohabiting men and women in the study sample (5.9% and 7.6%, respectively) are higher than those found in national studies in Tanzania (5.2% and 5.4%, respectively). However, they are very similar to those found in previous surveys which were held in Ifakara town (33).

In our study population, three of the investigated social drivers were significantly associated with differences in HIV status: gender norms (the belief that a woman cannot ask her husband to use a condom when she knows he has a disease), marriage characteristics (being remarried), and sexual behavior characteristics (number of lifetime sexual partners). Other investigated variables (sociodemographic characteristics, health care utilization, and lifestyle characteristics) were not associated with differences in HIV status.

Gender researchers have advocated the use of two separate concepts for investigating male versus female differences in health; biological sex and socially-constructed gender roles (38). Our study results highlight the relevance of this distinction. Not biological sex, but socially constructed gender norms, appeared to be associated with differences in HIV status in married individuals in the study area.

Our findings as regards to gender are credible in the light of other studies. Several studies have also found that, by limiting women's options for protecting themselves from HIV and other sexually transmittable infections, gender discriminatory practices can be a driver of the HIV epidemic (19–22). A study from Malawi suggested that gender inequality within marital relationships has a negative influence on HIV prevention (27).

Women's lower condom negotiation power is likely to be linked to broader societal norms about appropriate (sexual) behavior for men and women. It has been pointed out that women in SSA may feel restrained to discuss condoms with their stable partners (husbands), because the women who do so are likely to be perceived as overly interested in sex (39), distrustful of their male partners, or promiscuous (40).

A study from rural Tanzania found that religious norms can also strengthen male dominance in marital sexual relationships and that only single women are allowed to propose condom use (41).

In our study, 17% of the respondents were remarried. Our observation that remarriage is an independent risk factor for HIV among married individuals corroborates with findings from a previous survey study of the relationship between HIV and remarriage that was conducted among representative population samples in 13 countries in SSA. This study found high rates of remarriage in almost all countries, with significantly higher rates of HIV prevalence among remarried individuals than among those married only once (42).

Although we did not explore the type of remarriage arrangements in this study, we know from anecdotal evidence that the engagement in trial or ‘transient marriages’ is a common socially formalized and culturally acceptable practice in the study area.

People engage in such marriages for a specific period of time, with the intention of finding out if a spouse is appropriate or inappropriate for a subsequent long-term official marriage. This custom of trial marriages may not be well-reflected in the ‘official’ remarriage statistics we obtained in our study. Nevertheless, this custom should not be disregarded. As Greenwood (43) pointed out, individuals who engage in trial marriages may not be interested in testing for HIV before they make an official commitment to live as spouses. Given the high HIV-related mortality rates in the region, people who engage in subsequent marriages may include widows who were infected with HIV by their previous partners. A study among widows and widowers in Uganda found that the majority of them got remarried following the death of their spouses, even though they were aware of their own HIV status. This resulted in the infection of new sexual partners in ways that were considered unfair and outrageous by the authors (43), the available data from our study did not provide information to substantiate these findings, however.

It is often assumed that engaging in concurrent sexual relationships is a risk factor for HIV (44, 45). Yet, surprisingly, in our study concurrent sexual partnership was not significantly associated with HIV status. One possible explanation is that our study measured HIV prevalence and included people who have been living with HIV for many years. People who know they are HIV positive may be less likely to engage in concurrent sexual partnerships.

Similarly, we found that living in an official polygamous marriage was not a predictor for HIV. This corresponds with findings from a previous study by Reniers and Watkins (26). This study found that being in a polygamous marriage does not increase women's vulnerability for HIV. The researchers argued that the distinctive structure of sexual networks in polygamous marriages and the lower coital frequency in conjugal dyads may help prevent or delay HIV transmission between the partners involved.

However, in our study the number of respondents in polygamous marriages was low, and further evidence from Tanzania is needed to substantiate hypotheses about association between polygamy and HIV.

Our finding as regards to the positive association between the number of an individual's previous sexual relationships and HIV prevalence is not new. The same association has been found by Kalichman et al. (46) and in another study from Tanzania (47).

Sociodemographic indicators, such as income and education are regarded as important predictors of health in theoretical models of social determinants of health (14). It is quite surprising that none of the measured sociodemographic variables were independently associated with a differential vulnerability for HIV in our study population. Yet, it is known that the relationship between sociodemographic characteristics and HIV can be quite complex. For instance, in a study of the social determinants of HIV serostatus in SSA, Fox et al. (48) found an inverse relationship between poverty and acquisition of HIV. This was contrary to the expectation, but understandable given the wider socioeconomic context.

## Strengths and limitations

The strength of this study is that we were able to use reliable survey data and blood samples from a large community-based cohort study (MZIMA). However, there are also limitations. First, two- thirds of the married or cohabiting respondents in the MZIMA cohort are female, which means that our study sample is not representative of the male/female distribution in the study area. Second, whereas the MZIMA study was not specifically designed to investigate social drivers of HIV, the available dataset allowed us to analyze data on only a limited set of potential social drivers of HIV. Although the available data are relevant as such, more specific data, particularly as regards to gender norms, will be needed to gain a deeper understanding of the impact of social drivers on HIV in married couples.

Third, in our study, the data about the relationship between polygamous marriage and HIV status are inconclusive because the number of study participants who were living in official polygamous marriages was small. Further research, incorporating larger samples of individuals in polygamous relationships is needed in Tanzania, to provide information about the association between (legally sanctioned) polygamy and HIV. Finally, the information on the social drivers that is collected in the MZIMA survey is based on self-reports. The influence of culture and social desirability on self-reported data on social drivers such as gender norms and VCT utilization or alcohol use is unknown.

## Implications

We are entering a new HIV prevention era, whereby approaches focusing on the prevention of individual risk behaviors for HIV are now being expanded with structural approaches that aim to tackle the underlying social determinants that shape and sanction people's lives and (risk) behaviors (10–17, 23, 49). Individuals in married and cohabiting relationships are an important target group for HIV prevention activities in Tanzania. The present study suggests that, in addition to individual interventions, the development of structural interventions that address broader societal gender norms and social practices with respect to remarriage and multiple partnerships can be particularly relevant for HIV prevention programs for this target group. At the same time, however, it should be kept in mind that some of these norms and practices may be difficult to change, as they are deeply rooted in society and partly sanctioned by Tanzanian family laws (LMA) (31).

## Conclusions

In this community-based cross-sectional study, we found that social practices/drivers influencing differential HIV vulnerability in married and cohabiting couples in Ifakara, Tanzania include gender norms, remarriage, and the number of lifetime sexual partners. Contrary to the expectation, other social factors (e.g. wealth, education, being in a polygamous marriage) were not significantly associated with HIV status in the studied population. Our data provide baseline information for developing further research on social drivers of HIV and comprehensive HIV prevention programs for married couples.

## Supplementary Material

Prevalence and social drivers of HIV among married and cohabitating heterosexual adults in south-eastern Tanzania: analysis of adult health community cohort data
