# Supplementary material for: Prevalence and social drivers of HIV among married and cohabitating heterosexual adults in south-eastern Tanzania: analysis of adult health community cohort data
Source: Glob Health Action. 2015 Sep 30;8:10.3402/gha.v8.28941. doi: 10.3402/gha.v8.28941 (PMC4592501; doi:10.3402/gha.v8.28941)
Supplement: Prevalence and social drivers of HIV among married and cohabitating heterosexual adults in south-eastern Tanzania: analysis of adult health community cohort data [file GHA-8-28941-s001.pdf]

Additional File 1

Table 1a. Characteristics of the study population, by sex : frequency distribution (n = 3,737 **)

Variable	ALL		Male		Female	
	Number of all respondents	Percent (%)	Number of male respondents	Percent (%)	Number of female respondents	Percent (%)
**TOTAL**	**3,988**	**100.0**	**1,340**	**33.6**	**2,648**	**66.4**
Social and demographic characteristics						
Sex						
Male	1,340	33.6	1,340	100.0	NA	NA
Female	2,648	66.4	NA	NA	2,648	100.0
Age (years)*						
≤20	175	4.4	6	0.5	169	6.4
21-30	1,265	31.8	234	17.5	1,031	39.0
31-40	1,159	29.1	416	31.1	743	28.1
41-50	613	15.4	247	18.5	366	13.8
50+	770	19.3	434	32.5	336	12.7
Mean = 38.4, SD = 14.5, Min = 13, Max = 99						
Performs any income-generating activity						
Yes	2,909	72.9	1,211	90.4	1,698	64.1
No	1,079	27.1	129	9.6	950	35.9
Ever had formal education?						
Yes	3,494	87.6	1,245	92.9	2,249	84.9
No	494	12.4	95	7.1	399	15.1
Religion						
Muslim	1,559	39.1	531	39.6	1,028	38.8
Christian-Catholic	2,087	52.3	702	52.4	1,385	52.3
Other Christian	322	8.1	99	7.4	223	8.4
Other/none	20	0.5	8	0.6	12	0.5
Ethnic group						
Northern Western	307	7.7	113	8.4	194	7.3
Northern Eastern	133	3.3	59	4.4	74	2.8
Southern	3,548	89.0	1,168	87.2	2,380	89.9
Marital characteristics						
Re-married?						
Yes	674	16.9	251	18.7	423	16.0
No	3,314	83.1	1,089	81.3	2,225	84.0
Sexual behavior characteristics						
Life-time number of sexual partners*						
1	901	24.1	125	10.1	776	31.1
2-4	1,837	49.2	490	39.6	1,347	53.9
5+	999	26.7	624	50.4	375	15.0
Mean = 4.6, SD = 7.2, Min = 1, Max = 100						

Gender norms						
Woman's status						
Yes	3,454	86.6	1,158	86.4	2,296	86.7
No	534	13.4	182	13.6	352	13.3
Health care utilization						
Condom use at first sex*						
Yes	525	13.3	135	10.2	390	14.9
No	3,384	85.8	1,175	88.6	2,209	84.5
Don't know	33	0.8	17	1.3	16	0.6
Ever had VCT?						
Yes	1,008	25.3	270	20.2	738	27.9
No	2,980	74.7	1,070	79.9	1,910	72.1
Lifestyle characteristics						
Ever drank alcohol?						
Yes	1,103	27.7	582	43.4	521	19.7
No	2,885	72.3	758	56.6	2,127	80.3

* Missing data for some respondents; NA = Not Applicable

* * Currently married men and women who participated in MZIMA adult health community cohort conducted in Ifakara, Tanzania

Table 2a. Prevalence of HIV by background characteristics: Sex aggregated data (n =3,737 [A])

Variable	% HIV positive		
	All respondents	Male	Female
	n=3,988	n=1,340	n=2,648
**OVERALL**	**6.67**	**5.90**	**7.06**
Social demographic characteristics			
Age (years)			
≤20	4.57	16.67	4.14
21-30	6.72	5.13	7.08
31-40	7.42	5.77	8.34
41-50	7.01	5.67	7.92
50+	5.71	6.45	4.76
Performs any income-generating activity			
Yes	6.81	5.70	7.60
No	6.30	7.75	6.11
Ever had formal education?			
Yes	6.41*	5.62	6.85
No	8.50*	9.47	8.27
Religion			
Muslim	7.31	7.16	7.39
Christian-Catholic	6.23	4.70	7.00
Other Christian	6.52	7.07	6.28
Other/none	5.00	12.50	0.00
Ethnic group			
Northern Western	7.49	7.96	7.22
Northern Eastern	4.51	1.69	6.76
Southern	6.68	5.91	7.06
Marital characteristics			
Re-married?			
Yes	9.79***	8.76**	10.40**
No	6.04***	5.23**	6.43**
Sexual behavior characteristics			
Life-time number of sexual partners			
1	4.88*	7.20	4.51**
2-4	7.19*	6.33	7.50**
5+	7.21*	5.61	9.87**
Gender norms			
Woman's status			
Yes	6.40*	5.53	6.84
No	8.43*	8.24	8.52
Health care utilization			
Condom use at first sex			
Yes	6.10	8.89	5.13
No	6.86	5.70	7.47
Don't know	0.00	0.00	0.00
Ever had VCT?			
Yes	7.84*	5.19	8.81**
No	6.28*	6.07	6.39**

Lifestyle characteristics			
Ever drank alcohol?			
Yes	7.25	5.67	9.02*
No	6.45	6.07	6.58*

***P<0.001, **P<0.050, *P<0.100

[A] Currently married men and women who participated in MZIMA adult health community cohort sex- aggregated data
